# Prevalence of and Risk Factors for Dry Eye Symptom in Mainland China: A Systematic Review and Meta-Analysis

**DOI:** 10.1155/2014/748654

**Published:** 2014-10-15

**Authors:** Ning-ning Liu, Lei Liu, Jun Li, Yi-zhou Sun

**Affiliations:** ^1^The Department of Ophthalmology, The First Affiliated Hospital of China Medical University, Shenyang, Liaoning 110001, China; ^2^The Department of Cardiovascular Ultrasound, The First Affiliated Hospital of China Medical University, Shenyang, Liaoning 110001, China

## Abstract

*Purpose*. To evaluate the pooled prevalence rate and risk factors of dry eye symptoms (DES) in mainland China.* Methods*. All the published population-based studies investigating the prevalence of DES in China were searched and evaluated against inclusion criteria. A systematic review and meta-analysis were performed.* Results*. Twelve out of the 119 identified studies were included in the meta-analysis. The pooled prevalence of DES in China was 17.0%. Female individuals, subjects living in the Northern and Western China, and over 60 years of age had significantly higher prevalent rates (21.6%, 17.9%, 31.3%, and 34.4%, resp.) compared with their counterparts. Patients with diabetes were also found to be more vulnerable to DES.* Conclusions*. The pooled prevalence rate of DES in mainland China was lower than that in other Asian regions and countries. A remarkable discrepancy in the prevalence in different geographic regions was noted. Aging, female gender, and diabetes were found to be risk factors for DES in China.

## 1. Introduction

Dry eye disease (DES) is a major tear deficiency disorder which causes discomfort, visual disturbances, and tear film instability with potential damage to the ocular surface [1]. The tear film and ocular surface form a complex and stable system that can lose its equilibrium through multiple disturbing factors [2]. Despite the gain in knowledge of pathogenic factors of DES acquired in the past decades, there has been considerable discrepancy in the reported prevalence worldwide, mainly due to lack of consensus on appropriated diagnostic criteria and differences in the parameters and research methodology applied [2]. Two large population-based studies suggested that about 7.8% of American women and 4.7% of men aged 50 years and older had DES [3, 4]. In a study conducted in Melbourne, Australia [5], DES was diagnosed in subjects over 40 years old as 10.8% by rose Bengal staining, 16.3% by Schirmer's test, 8.6% by tear breakup time, 7.4% with two or more signs, and 5.5% with severe symptoms of DES not attributed to hay fever. Women were more likely to report severe symptoms. Risk factors for two or more signs included age and self-report of arthritis. A large study used questionnaires to investigate the prevalence of DES in Canada in all age groups [6]. In the 13,517 returned questionnaires (55% aged 21–50 years, 60.7% were women), 28.7% respondents reported DES. Those reporting severe DES were predominantly women, with a ratio of 46 : 1.

Some population-based studies investigating the prevalence and risk factors of DES in China have been published [7–18]. These studies, however, were all based on regional population. Meanwhile, the number of regions with reported data is limited. Vast regions in China have no published epidemiologic data on DES. Therefore, it is difficult to assess the overall prevalence of DES in China. Based on the current published data, the present study utilized systematic review and meta-analysis with an aim to estimate the overall pooled prevalence of DES in China. The epidemic characteristics of DES such as prevalence in different geographic regions, genders, and age groups were also explored.

## 2. Methods

### 2.1. Search Strategy and Inclusion Criteria

To obtain regional epidemiologic data, we searched Medline (from January 1, 1946, to October 31, 2013), Embase (from January 1, 1950, to October 31, 2013), the Cochrane Central Register of Controlled Trials (up to 2013, issue 10), Chinese Biological Medicine (January 1, 1978, to October 31, 2013), China National Knowledge Infrastructure (January 1, 1979, to October 31, 2013), Wan Fang Data (January 1, 1982, to October 31, 2013), and Chongqing VIP database (January 1, 1982, to October 31, 2013) using the free combinations of the terms “Dry Eye,” “prevalence,” and “China” in both English and Chinese languages. Reference lists were checked. The corresponding authors or first authors of the publications were contacted if additional information was needed, results were unclear, or relevant data were not reported.

The review and analysis were guided to be conducted by the PRISMA statement for preferred reporting of systematic reviews and meta-analysis [19]. Reports potentially eligible for inclusion in this systematic review and meta-analysis had to meet the following criteria: population-based studies, authentic, written either in English or in Chinese, and being able to provide sufficient information to estimate the pooled prevalence of and risk factors for DES. Population-based study needed to meet the following criteria: (i) the study populations need to be scrutinized with regard to all characteristics of the cohort before one can compare their results and (ii) the study populations need to be from special samples except hospital. If more than one study was based on the same population sample, the study with better quality was included. We excluded studies that were on the duplicate population groups but of lower quality and those that had participants drawn from a particular occupation or population and those that did not satisfy one or more inclusion criteria.

### 2.2. Data Extraction and Quality Assessment

The literatures were searched independently by two researchers (Lei Liu and Yi-zhou Sun). Data were extracted from each article using a standardized form which included the first author, publication year, region or province, age range, sample size, diagnostic criteria, prevalence definition, response rage, and quality of the studies (in a score of 1–5) (Table 1).

### 2.3. Statistical Analysis

Odds ratio (OR) was analyzed using RevMan version 5.0 (Review Manager, Copenhagen: The Nordic Cochrane Centre, The Cochrane Collaboration, 2010) statistical software package. Multivariate analyses to test the individual association of each variable with the overall pooled prevalence estimate using meta-regression analyses were performed by Comprehensive Meta-Analysis software version 2.0 (Biostat, Englewood Cliffs, NJ, USA). All meta-analyses were evaluated for heterogeneity using the Chi-square based *I* ^2^ test and *Q* test [20]. *I* ^2^ estimated the percentage of the total variance in all of the data under consideration that was related to heterogeneity. If a moderate or high level heterogeneity was observed, random-effects meta-analysis was performed by the DerSimonian and Laird method, except when using fixed-effects models. Assessment of publication bias was done by inspecting a funnel plot and using Egger's and Begg's test. *P* < 0.05 was considered statistically significant [21, 22].

## 3. Results

**Figure 1:**
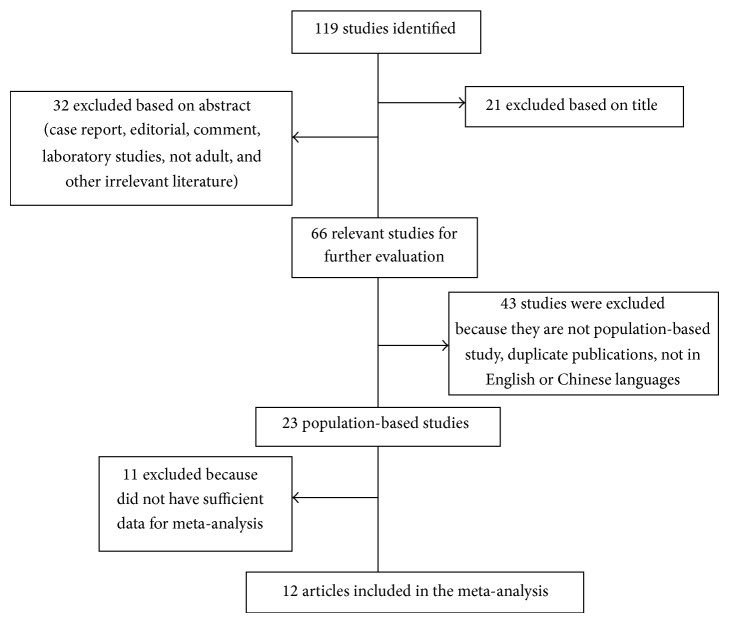
Flow chart demonstrating how the identified published studies were included in the meta-analysis.

The flow chart showing how the identified published studies were included in the meta-analysis was demonstrated in Figure 1. We identified 119 potentially relevant articles through electronic and hand searches. After systematic review, only 12 studies [7–18] (Table 1) met the inclusion criteria and were included in the meta-analysis.

**Figure 2:**
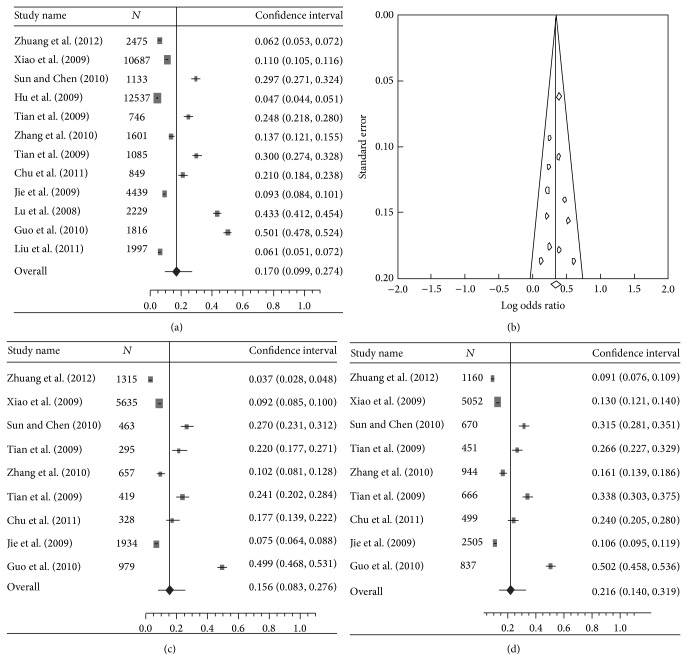
(a) A forest plot displaying the pooled prevalence of dry eye syndromes (DES) in the population of mainland China. (b) A funnel plot of studies conducted on the prevalence of DES in China. (c) A forest plot displaying the pooled prevalence of DES in the male gender in China. (d) A forest plot displaying the pooled prevalence of DES in the female gender in China.

**Figure 3:**
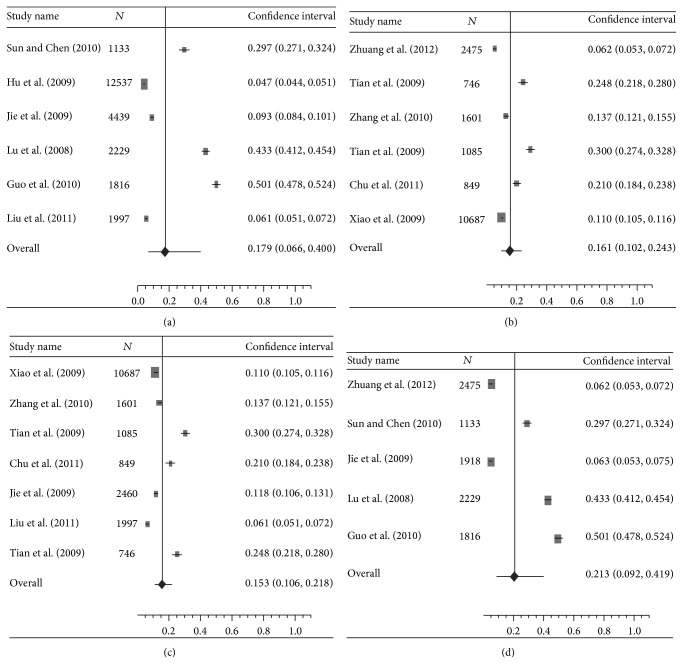
(a) A forest plot displaying the pooled prevalence of DES in individuals living in Northern China. (b) A forest plot displaying the pooled prevalence of DES in individuals living in Southern China. (c) A forest plot displaying the pooled prevalence of DES in individuals living in urban China. (d) A forest plot displaying the pooled prevalence of DES in individuals living in rural China.

**Figure 4:**
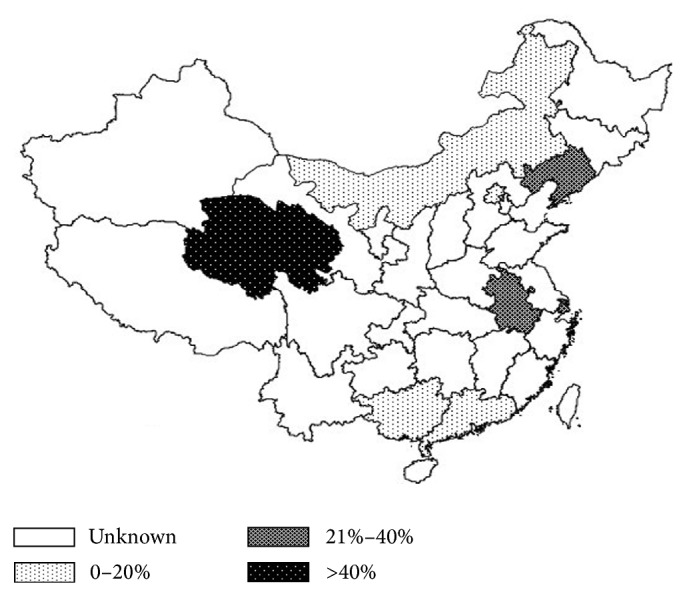
A map displaying the prevalence rates and geographic locations in China from 12 studies included in the meta-analysis.

The pooled prevalence of DES was 17.0% (95% CI: 9.9%–27.4%) (*I* ^2^ = 49.9%,  *Q* = 0.99,  *P* < 0.001) in overall population (Figures 2(a) and 2(b), Table 2). The pooled prevalence rate in female individuals was significantly higher than that in males (21.6% versus 15.6%) (OR: 1.41, 95% CI: 1.31–1.52, and *P* < 0.001) (Figures 2(c) and 2(d), Table 2). No significant difference was found in prevalence rate between urban China and rural China (15.3% and 21.3%, resp., OR: 1.06, 95% CI: 0.97–1.17, and *P* = 0.205) (Figures 3(c) and 3(d), Table 2). The prevalence rates, however, were found to be remarkably variable in different geographic regions in China. Northern China had significantly higher prevalence than Southern China (17.9% versus 16.1%) (OR: 1.97, 95% CI: 1.78–2.04, and *P* < 0.001) (Figures 3(a) and 3(b), Table 2). Compared with Central China which had a prevalence rate of 10.3%, Eastern China and Western China showed significantly higher prevalence rate (12.8% and 31.3%, resp.) (OR: 2.62 and 1.38, resp., and *P* < 0.001) (Table 2). The meta-analysis data covered eight provincial/municipality/autonomous regions as shown in Figure 4.

Five studies investigated the association between age and DES. The pooled OR was 3.34 (95% CI, 2.68–4.16) (*I* ^2^ = 15%,  *P* < 0.001) for elder age (≥60 years). Seven studies explored the relationship between the prevalence of DES and genders. The pooled OR was 1.15 (95% CI, 1.04–1.27) (*I* ^2^ = 12%,  *P* = 0.005) for female gender. In addition, diabetes also showed a significant risk factor for DES and the pooled OR was 3.82 (95% CI, 2.68–5.46) (*I* ^2^ = 10%,  *P* < 0.001). The results are presented in Table 3.

All comparisons passed the test of heterogeneity, as previously defined. Random-effects models were used for meta-analysis. There was no significant publication bias in this meta-analysis (*P* < 0.05 for both Egger's test and Begg's test).

## 4. Discussion

To our knowledge, the present study is the first meta-analysis of DES in mainland China. Our study showed a pooled prevalence rate of DES at 17%. Population-based studies evaluating the prevalence of DES differ in the definitions, diagnostic criteria for DES, selection of the study population, and the methodology applied (questionnaires and/or objective tests). Comparisons between the studies are hence difficult [2]. Not surprisingly, there is a discrepancy in the prevalence between our data and findings in other Asian regions and countries. The Shihpai Eye Study found that 33.7% (459/1361) of individuals aged ≥65 years in Taiwan were symptomatic, as defined by the reporting of one or more dry eye symptoms often or at all times [23]. In Yongin, Korea, the adjusted prevalence of dry eye disease was 33.2% in 657 subjects aged 65 years or older [24]. A Japanese study found that 21.6% of the female individuals aged 40 years or over were diagnosed with dry eye disease or severe symptoms, significantly higher than their male counterparts (12.5%) [25].

Report suggested that the prevalence of DES increased with age [26], which is consistent with our meta-analysis. Our findings verified that the elder population (over 60 years of age) had higher prevalence of DES than the younger population. Women are reported to be particularly susceptible to DES [27, 28]. However, some published studies based on regional population in China did not find any prevalence difference between males and females [7, 13–17]. The conflicting findings may be caused by the difference in the selection of population. The pooled prevalence in our study showed significantly higher prevalence in female subjects, consistent with majority of studies.

A larger number of DES cases were identified in individuals with diabetes, particularly those with type 2 diabetes [29]. Our meta-analysis illustrated significantly higher prevalence of DES in diabetes patients, which is consistent with other studies [30, 31]. The prevalence of diabetes has been increased dramatically with the rapid economic growth and the improved quality of life in China over the last thirty years [32]. The prevalence of DES is expected to rise further as a result. Much attention should be paid to prevent ocular surface disorder in the people with diabetes.

Other risk factors for DES (e.g., alcohol, smoking, computer use, contact lens wear, and systemic or ocular medications) were initially included in the present study but were excluded because the pooled OR was not able to be calculated as a result of insufficient information provided.

Our findings revealed remarkable difference in DES prevalence in different geographic regions in China. Western China and Northern China had significantly higher prevalence when compared with Central, Eastern, and Southern China, possibly because of the difference in the climate conditions and geographic characteristics in these regions. Previous study suggested there was a significant relationship between ultraviolet radiation and dry eye [33]. Qinghai-Tibetan Plateau of Western China is characterized with high altitudes, long hours of sunlight, and strong ultraviolet radiation [34], which may contribute to the high prevalence. Other climate conditions including low humidity and draft were believed to be related to DES [35], which may partially be an explanation of the higher prevalence in Northern China. Although the pooled prevalence in rural China seemed to be higher than in urban China, the difference was not statistically significant after meta-regression. Further investigation is needed.

Because the findings in pooled prevalence of DES had moderate *I* ^2^, we did meta-regression to analyze and verify the results to avoid substantial heterogeneity. Although pooled prevalence of DES in mainland China was derived in the present study, there are some limitations. Firstly, the pooled prevalence data was estimated using meta-analysis, rather than prevalence in a single national population-based study. Secondly, China is a vast country geographically with 34 provincial-level administrative units (23 provinces, 4 municipalities, 5 autonomous regions, and 2 special administrative regions). Our study only included data from 12 units, which may be inaccurate to represent the pooled prevalence of the whole country. More regional epidemiologic studies are warranted, particularly those units with no published data, so that a more accurate picture of prevalence in China can be drawn. Lastly, our data should be updated as a result of the emerging new data.

## 5. Conclusions

Compared with some of the other Asian regions and countries, the pooled prevalence of DES in mainland China was lower. There is remarkable discrepancy in the prevalence in different geographic regions in China, with Western and Northern China presenting higher prevalence, possibly because of the difference in the climate conditions and geographic characteristics. Female, elder individuals, and patients with diabetes seemed to be more vulnerable to DES. More studies focusing on Chinese populations in regions without epidemiologic data are of great value.

## Figures and Tables

**Table 1:** Characteristics of population-based studies on the prevalence of DES.

Article number	First author, publication year	Province	Geographical area (latitude-longitude)	Rural/urban	Age range (year)	Sample size (*N*)	Case (*n*)	Checklist	Diagnosis standard	Sampling scheme	Prevalence definition	Response rate (%)	Total score
1	Zhuang 2012 [7]	Guangdong	Southern-Eastern	Rural	14–90	2475	154	(1) signs, (2) BUT, (3) FL, (4) SIT	(1) combined with two of (2–4)	yes	yes	88.39	5
2	Xiao 2009 [8]	Guangxi	Southern-Western	Urban	over 20	10687	1179	(1) signs, (2) BUT, (3) FL, (4) SIT	(1) combined with two of (2–4)	yes	yes	92.04	5
3	Sun 2010 [9]	Liaoning	Northern-Eastern	Rural	20–80	1133	336	(1) signs, (2) BUT, (3) FL, (4) SIT	(1) combined with two of (2–4)	yes	no	N/A	3
4	Hu 2009 [10]	Inner Mongolia	Northern-Central	Rural/urban	over 40	12537	595	(1) signs, (2) BUT, (3) FL, (4) SIT	(1) combined with two of (2–4)	yes	no	N/A	3
5	Tian 2009 [11]	Shanghai	Southern-Eastern	Urban	60–90	746	185	(1) signs, (2) BUT, (3) FL, (4) SIT	(1) combined with two of (2–4)	yes	yes	93.25	5
6	Zhang 2010 [12]	Shanghai	Southern-Eastern	Urban	45–75	1601	219	(1) signs, (2) BUT, (3) FL, (4) SIT	(1) combined with two of (2–4)	yes	yes	N/A	4
7	Tian 2009 [13]	Shanghai	Southern-Eastern	Urban	20–95	1085	326	(1) signs, (2) BUT, (3) FL, (4) SIT	(1) combined with two of (2–4)	yes	yes	85.7	5
8	Chu 2011 [14]	Anhui	Southern-Central	Urban	50–95	849	178	(1) signs, (2) BUT, (3) FL, (5) SIT	(1) combined with two of (2–5)	yes	yes	90.68	5
9	Jie 2009 [15]	Beijing	Northern-Eastern	Rural/urban	40–101	4439	411	(1) signs, (2) BUT, (3) FL, (5) SIT	(1) combined with two of (2–5)	yes	yes	83.4	5
10	Lu 2008 [16]	Qinghai	Northern-Western	Rural	over 40	2229	965	(1) signs, (2) BUT, (3) FL, (5) SIT	Significant symptoms for dry eye	yes	yes	84.69	5
11	Guo 2010 [17]	Qinghai	Northern-Western	Rural	40–91	1816	909	(1) signs, (2) BUT, (3) FL, (5) SIT	Significant symptoms for dry eye	yes	yes	84.9	5
12	Liu 2011 [18]	Beijing	Northern-Eastern	Urban	20–90	1997	122	(1) signs, (2) BUT, (3) FL, (5) SIT	(1) combined with two of (2–5)	no	yes	N/A	3

BUT: break-up time; NA: not available; CI: confidence interval; SIT: Schirmer I test; FL: fluorescein score.

**Table 2:** The pooled prevalence of DES in mainland China with different populations and regions.

Variable	Number of articles	Case/total	Pooled estimate [95% CI] (%)	Heterogeneity *I* ^2^ (%)∗	*Q* value	OR [95% CI]	*P* value
General population	12	5579/41594	17.0 [9.9–27.4]	49.9	0.99		
Gender							
Male	9	1619/12025	15.6 [8.3–27.6]	49.8	0.99	1.00	
Female	9	2278/12784	21.6 [14.0–31.9]	49.8	0.99	1.41 [1.31–1.52]	<0.001
Rural/urban							
Rural	5	2485/9571	21.3 [9.2–41.9]	49.9	0.99	1.00	
Urban	7	2499/19425	15.3 [10.6–21.8]	49.7	0.99	1.06 [0.97–1.17]	0.205
Northern/Southern							
Northern	6	3338/24151	17.9 [6.6–40.0]	49.7	0.99	1.97 [1.78–2.04]	<0.001
Southern	6	2241/17443	16.1 [10.2–24.3]	49.8	0.98	1.00	
Age							
≤60 yrs	12	2294/14996	14.2 [8.7–22.3]	49.8	0.99	1.00	
>60 yrs	12	1856/6904	34.4 [23.7–47.1]	49.6	0.98	3.49 [3.12–3.84]	<0.001
Area							
Eastern	7	1753/13476	12.8 [7.3–21.4]	49.7	0.98	2.62 [2.32–2.95]	<0.001
Central	2	773/13386	10.3 [2.2–37.1]	49.8	0.99	1.00	
Western	3	3053/14732	31.3 [10.1–65.0]	49.8	0.99	1.38 [3.39–11.47]	<0.001

CI: confidence interval. ORs: odds ratios. ^*^ *P* < 0.001.

**Table 3:** The pooled odds ratio for risk factors of DES.

Variable	Pooled odds ratio	95% CI (%)		*P* value
		Lower limit	Upper limit	
Females	1.15	1.04	1.27	0.005
Age	3.34	2.68	4.16	<0.001
Diabetes	3.82	2.68	5.46	<0.001

CI: confidence interval.

## References

[1] Ding J., Sullivan D. A. (2012). Aging and dry eye disease. *Experimental Gerontology*.

[2] Brewitt H., Sistani F. (2001). Dry eye disease: the scale of the problem. *Survey of Ophthalmology*.

[3] Schaumberg D. A., Sullivan D. A., Buring J. E., Dana M. R. (2003). Prevalence of dry eye syndrome among US women. *The American Journal of Ophthalmology*.

[4] Schaumberg D. A., Dana R., Buring J. E., Sullivan D. A. (2009). Prevalence of dry eye disease among US men: estimates from the physicians' health studies. *Archives of Ophthalmology*.

[5] McCarty C. A., Bansal A. K., Livingston P. M., Stanislavsky Y. L., Taylor H. R. (1998). The epidemiology of dry eye in Melbourne, Australia. *Ophthalmology*.

[6] Caffery B. E., Richter D., Simpson T., Fonn D., Doughty M., Gordon K. (1998). CANDEES. The Canadian Dry Eye Epidemiology Study. *Advances in Experimental Medicine and Biology*.

[7] Zhuang S.-J., Lei S.-C., Luo X.-D., Wang D.-L., Wen J.-J., Deng D.-W. (2012). Epidemiologic survey of dry eye in a community of Huidong County in Guangdong province. *Chinese Journal of Experimental Ophthalmology*.

[8] Xiao X. L., Wei F. B., Wei L. Y., Xu J. Z. (2009). Epidemiologic investigation and study of dry eye in common crowd of Liuzhou. *International Journal of Ophthalmology*.

[9] Sun Y. Z., Chen L. (2010). Preliminary incidence of dry eye in coastal rural and urban residents of Liaoning. *Shandong Yi Yao*.

[10] Hu L. X., Zhang Y. Q., Nie Q., Wang M., Jia C. R., Du Z. Y. (2009). Investigation of dry eye prevalence rate in InnerMongolia normal people above 40 years. *Journal of Clinical Ophthalmology*.

[11] Tian Y. J., Liu Y., Zou H. D., Fu J., Shen B. J., Wang W. W., Xu X. (2009). The epidemiologic study of dry eye in Beixinjing district of Shanghai. *Chinese Journal of Practical Ophthalmology*.

[12] Zhang Y., Ge L., Huang H. L., Ni J. P., Wu C. R., Fu C. W. (2010). Investigation and analysis of dry eye prevalence middle-ged and senior residents in Huamu community of Shanghai. *Practical Clinical Medicine*.

[13] Tian Y. J., Liu Y., Zou H. D., Jiang Y. J., Liang X. Q., Sheng M. J., Li B., Xu X. (2009). Epidemiologic study of dry eye in populations equal or over 20 years old in Jiangning District of Shanghai. *Zhonghua Yan Ke Za Zhi*.

[14] Chu Z. D., Yao Y., Fu D. H. (2011). Prevalence of dry eye in populations equal or over 50 years old in Helie districts of Wuxi. *Chinese Journal of Practical Ophthalmology*.

[15] Jie Y., Xu L., Wu Y. Y., Jonas J. B. (2009). Prevalence of dry eye among adult Chinese in The Beijing Eye Study. *Eye*.

[16] Lu P., Chen X., Liu X., Yu L., Kang Y., Xie Q., Ke L., Wei X. (2008). Dry eye syndrome in elderly Tibetans at high altitude: a population-based study in China. *Cornea*.

[17] Guo B., Lu P., Chen X., Zhang W., Chen R. (2010). Prevalence of dry eye disease in Mongolians at high altitude in China: The Henan Eye Study. *Ophthalmic Epidemiology*.

[18] Liu Y., Zou L. H., Zhao M., Jia L. Y., Zhu J. B. (2011). Investigation of dry eye prevalence rate in Beijing Xicheng district. *Chinese Journal of Practical Ophthalmology*.

[19] Moher D., Liberati A., Tetzlaff J., Altman D. G., The PRISMA Group (2009). Preferred reporting items for systematic reviews and meta-analyses: the PRISMA statement. *PLoS Medicine*.

[20] Higgins J. P. T., Thompson S. G., Deeks J. J., Altman D. G. (2003). Measuring inconsistency in meta-analyses. *British Medical Journal*.

[21] Mantel J., Haenszel W. (1959). Statistical aspects of the analysis of data from retrospective studies of disease. *Journal of the National Cancer Institute*.

[22] der Simonian R., Laird N. (1986). Meta-analysis in clinical trials. *Controlled Clinical Trials*.

[23] Lin P.-Y., Tsai S.-Y., Cheng C.-Y., Liu J.-H., Chou P., Hsu W.-M. (2003). Prevalence of dry eye among an elderly Chinese population in Taiwan: The Shihpai Eye Study. *Ophthalmology*.

[24] Han S. B., Hyon J. Y., Woo S. J., Lee J. J., Kim T. H., Kim K. W. (2011). Prevalence of dry eye disease in an elderly Korean population. *Archives of Ophthalmology*.

[25] Uchino M., Nishiwaki Y., Michikawa T., Shirakawa K., Kuwahara E., Yamada M., Dogru M., Schaumberg D. A., Kawakita T., Takebayashi T., Tsubota K. (2011). Prevalence and risk factors of dry eye disease in Japan: Koumi Study. *Ophthalmology*.

[26] Smith J. A., Albenz J., Begley C., Caffery B., Nichols K., Schaumberg D., Schein O. (2007). The epidemiology of dry eye disease: report of the epidemiology subcommittee of the International Dry Eye Workshop. *Ocular Surface*.

[27] Galor A., Feuer W., Lee D. J., Florez H., Carter D., Pouyeh B., Prunty W. J., Perez V. L. (2011). Prevalence and risk factors of dry eye syndrome in a United States Veterans affairs population. *The American Journal of Ophthalmology*.

[28] Gayton J. L. (2009). Etiology, prevalence, and treatment of dry eye disease. *Clinical Ophthalmology*.

[29] Najafi L., Malek M., Valojerdi A. E., Aghili R., Khamseh M. E., Fallah A. E., Tokhmehchi M. R. F., Behrouz M. J. (2013). Dry eye and its correlation to diabetes microvascular complications in people with type 2 diabetes mellitus. *Journal of Diabetes and its Complications*.

[30] Seifart U., Strempel I. (1994). The dry eye and diabetes mellitus. *Ophthalmologe*.

[31] Moss S. E., Klein R., Klein B. E. K. (2008). Long-term incidence of dry eye in an older population. *Optometry and Vision Science*.

[32] Yang W., Lu J., Weng J. (2010). China National Diabetes and Metabolic Disorders Study Group. Prevalence of diabetes among men and women in China. *The New England Journal of Medicine*.

[33] Backman H. A. (1982). The effects of PUVA on the eye. *American Journal of Optometry and Physiological Optics*.

[34] Wang Y., Yu J., Gao Q., Hu L., Gao N., Gong H., Liu Y. (2012). The relationship between the disability prevalence of cataracts and ambient erythemal ultraviolet radiation in China. *PLoS ONE*.

[35] Wolkoff P. (2010). Ocular discomfort by environmental and personal risk factors altering the precorneal tear film. *Toxicology Letters*.

